# Management of Patients with Graves' Disease and Orbital Involvement: Role of Spectral Domain Optical Coherence Tomography

**DOI:** 10.1155/2018/1454616

**Published:** 2018-02-18

**Authors:** Alice Bruscolini, Maurizio La Cava, Magda Gharbiya, Marta Sacchetti, Lucia Restivo, Chiara Nardella, Marco Marenco, Alessandro Lambiase

**Affiliations:** Department of Sense Organs, University Sapienza, Rome, Italy

## Abstract

**Purpose:**

To investigate the role of choroidal thickness evaluation with spectral domain optical coherence tomography (SDOCT) and enhanced depth imaging (EDI) technique in the management of patients with Graves' disease and orbitopathy (GO).

**Methods:**

Thirty-six eyes of 18 patients with GO and 36 eyes of 18 age-matched control subjects were included in this retrospective observational study. All the subjects underwent a complete ophthalmological evaluation, including clinical activity score (CAS) and exophthalmometry. The SDOCT images of the choroid were obtained by EDI modality.

**Results:**

Choroidal thickness was significantly increased in GO than in control eyes (*p* < 0.01). A significant correlation was found between choroidal thickness and CAS, proptosis, and the duration of disease (*p* < 0.05).

**Conclusion:**

This study shows that choroidal thickness, evaluated with EDI-OCT, is significantly increased in patients with GO and correlates with the activity of the disease, proptosis, and duration of the disease. The choroidal thickening may reflect the ocular hemodynamic changes, and enhanced depth imaging optical coherence tomography may be a useful tool for the evaluation of orbital congestion and management of patients with Graves' disease and orbital involvement.

## 1. Introduction

Graves' disease represents the most common cause of hyperthyroidism in adults. Orbital involvement is known as Graves' orbitopathy (GO) [1, 2]. The pathogenetic mechanisms of GO have not yet been fully resolved [3]. It is known that antibodies against the thyroid-stimulating hormone (TSH) receptors play an important role. TSH receptors can not only be found in the thyroid but also in the extraocular eye muscles and retrobulbar fat tissues. It is thought that circulating TSH receptor autoantibodies (TRAbs) trigger inflammation and activation of orbital fibroblasts leading to intraorbital swelling in an early active stage and, subsequently, to fibrosis at a later stage [4–6]. Active GO is characterized by an inflammatory response which may involve the ocular surface, extraocular muscles, and other orbital tissue. Depending on the site of inflammation, the disease may cause dry eye symptoms or conjunctival chemosis, while the increased orbital volume may cause proptosis and eye movement disorders [2, 7].

The pathogenesis of the acute inflammatory stage has been attributed to autoimmunity, but a number of clinical and experimental studies suggest that superior orbital vein congestion also plays an important role in the disease inflammatory staging and contributes to the development of clinical signs and symptoms (e.g., proptosis, muscle restriction, periorbital swelling, and chemosis) during the active stage of the disease [8–10]. These hemodynamic changes in orbital vessels can be observed by the orbital color Doppler examination; however, it is rarely performed, due to its poor reproducibility and repeatability [8–11]. As a consequence, novel diagnostic techniques able to assess orbital congestion are highly sought after. A prompt diagnosis and staging of disease activity and severity are mandatory to drive therapeutic approach and standardized management of GO. Recent evidence suggests that spectral domain optical coherence tomography (SDOCT) examination may represent a useful, safe, and rapid diagnostic tool to evaluate GO activity [12, 13]. SDOCT has been recently developed to assess retinal and optic nerve morphology and to quantify thickness of choroidal vascularization by using the enhanced depth imaging (EDI) method [14]. The choroid is primarily a vascular structure that supplies oxygen and nutrients to the outer retina. The choroidal veins drain in the ophthalmic veins and are devoid of the valve; therefore, systemic conditions that affect blood flow in the ophthalmic veins may influence the choroidal thickening [15]. Several evidence showed changes of choroidal thickness during physiological variations and in a wide range of systemic conditions including inflammatory and vascular diseases such as Vogt Koyanagi Harada, Behçet syndrome, sarcoidosis, and migraine [16, 17].

The aim of this study is to evaluate changes of choroidal thickness in patients with Graves' disease and orbitopathy and their relationship with clinical features and activity of the disease.

## 2. Patients and Methods

### 2.1. Study Design

Eighteen consecutive patients with diagnosis of Graves' disease and orbitopathy were included in this retrospective study at the Department of Sense Organs of the University Sapienza of Rome. Eighteen healthy, age-matched volunteers were enrolled among the unaffected companions of patients attending the outpatients' service of the Eye Clinic of the University of Rome “Sapienza”. Informed consent was obtained from all subjects involved in the study and the Local Ethics Committee approved the experimental protocol. The research followed the tenets of the Declaration of Helsinki.

**Figure 1:**
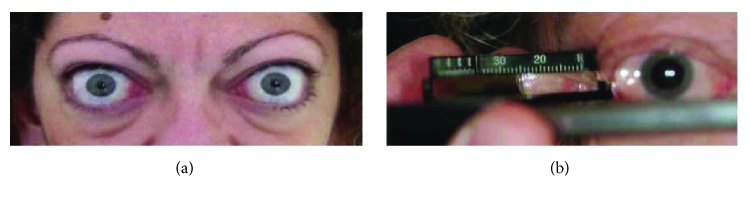
Patient with Graves' disease and orbitopathy showing a severe proptosis (a) as assessed by Hertel exophthalmometry (b).

All subjects were previously examined by an endocrinologist, and the laboratory diagnosis of Graves' disease was based on the finding of undetectable serum TSH and high blood thyroid hormone associated with the presence of circulating antibodies (TRAb). Clinical history was collected, and all patients underwent a complete eye examination including (i) exophthalmometry with Hertel instrument (Figure 1), (ii) measurement of eyelid aperture, (iii) assessment of subjective diplopia using Gorman score (0: no diplopia, 1: intermittent diplopia, 2: inconstant diplopia, and 3: constant diplopia) [7], (iv) measurement of visual acuity, (v) assessment of corneal status, (vi) fundus examination, (vii) ocular biometry (IOL Master, Carl Zeiss Meditec, Dublin, CA), and (viii) OCT.

Patients were included if they met the following criteria: (i) age 18 years or more, (ii) diagnosis of Graves' disease in the last 12 months, (iii) euthyroid in treatment with antithyroid drugs, and (iv) first episode of GO. All patients and controls included in this study did not use systemic steroids. To ensure reliable choroidal thickness assessment by SDOCT-EDI, all women were evaluated in the first week after menstruation and all patients with conditions associated with choroidal changes were excluded including refractive error > ± 3 spherical equivalent; axial length < 22 mm and >26 mm; intraocular pressure > 18 mmHg, cup/disc ratio > 0.5; any other systemic disease; any other ocular disease, such as glaucoma, uveitis, or central serous chorioretinopathy; history of uveitis or central serous chorioretinopathy; previous intraocular surgery; use of topical medication or systemic therapy with known interference on choroidal thickness such as steroids and diuretics; and low quality (<20 units) OCT images.

The activity of GO was assessed through the clinical activity score (CAS), [18, 19]. According to the EUGOGO (European Group of Graves' Orbitopathy) guidelines, patients were divided into nonactive GO (CAS < 3) and active GO (CAS ≥ 3). The severity of GO was also assessed based on these guidelines and rated as mild, moderate to severe, and sight threatening (very severe).

### 2.2. Optical Coherence Tomography

**Figure 2:**
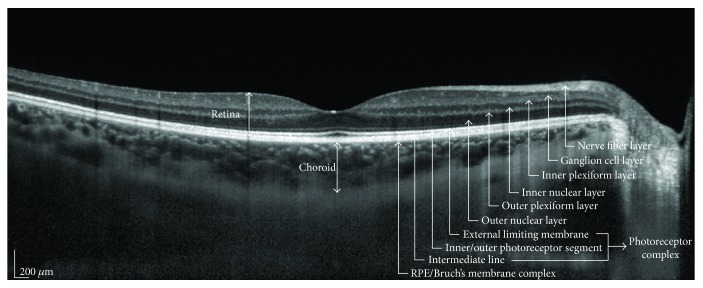
Optical coherence tomography scan showing the retinal layers and the macular choroidal thickness in a normal eye.

Included patients and controls underwent optical coherence tomography (OCT) examination using the Heidelberg Spectralis (Spectralis Family Acquisition Module, V 5.1.6.0; Heidelberg Engineering, Heidelberg, Germany) with Heidelberg Eye Explorer (V 1.7.1.0; Heidelberg). The SDOCT images of the choroid were obtained by enhanced depth imaging modality, following a standardized protocol described elsewhere [20]. In brief, two high-quality 30° horizontal and vertical line scans through the fovea with 90 to 100 frames averaged for each scan were obtained for each eye and the image with the best visualization of the border between the choroid and sclera was used. Choroidal thickness was measured using the manual caliper tool provided with the software of the OCT device. The choroid was defined as the layer between the base of the RPE and the hyperreflective line or margin corresponding to the chorioscleral interface (Figure 2).

The subfoveal choroidal thickness from the horizontal and vertical line scans was measured by 2 physicians (Magda Gharbiya and Lucia Restivo) who were masked to the subjects' diagnosis, and values were averaged.

### 2.3. Statistical Analysis

Statistical analysis was performed with the SPSS for Windows (V 17.0, SPSS, Chicago, IL, USA). Normal distribution of data was analyzed by the Kolmogorov–Smirnov test. Parametric variables were compared using the unpaired *t*-test. Levene's test was used to verify variance homogeneity. Nonparametric distributed values were analyzed by the Mann–Whitney *U* rank sum test. Categorical variables were compared using Fisher's exact test. OCT measurements between groups were compared using the general linear model, including age, gender, axial length, and smoking as covariates. Interobserver repeatability for choroidal thickness measurements was tested with the intraclass test/retest correlation. We followed the methods of Häner et al. [21].

Bivariate relationships were evaluated by the Spearman coefficient or the Pearson analysis as appropriate. Data are reported as mean values ± standard deviation. *p* values of less than 0.05 were considered as statistically significant.

## 3. Results

Thirty-six eyes of 18 patients (mean age, 44.1 ± 9.8 years; range, 24 to 57 years; 10 women and 8 men) with a diagnosis of GO and 36 eyes of 18 age-matched control subjects (mean age, 44.2 ± 10.7 years; range, 26 to 60 years; 11 women and 7 men; *p* > 0.05 for age and sex) were consecutively included in this study.

Demographic and clinical characteristics of the patients with GO and control subjects are summarized in Table 1.

In the patients' group, the mean duration of Graves' disease was 8.9 ± 2.0 (range, 5 to 12 months). Eight (55.6%) out of 18 patients had diplopia. The exophthalmometry measurements ranged from 14 to 26 mm (mean ± SD, 20.1 ± 3.6 mm). According to the severity assessment, 10 (44.4%) patients had a mild GO, and 8 patients had a moderate to severe disease. No patient showed a sight-threatening GO. The CAS score was <3 in 10 patients and ≥3 in the remaining 8 patients.

**Figure 3:**
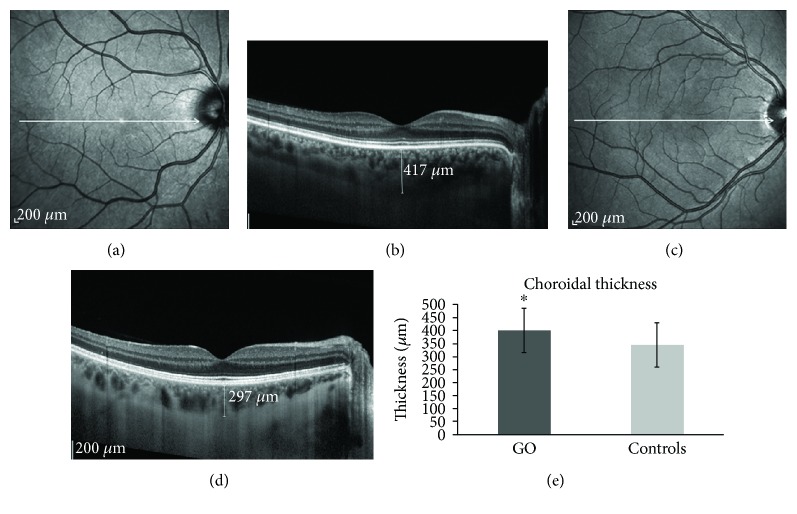
Subfoveal choroidal thickness measured by EDI SDOCT in a patient with Graves' orbitopathy (a, b) and in a healthy control (c, d). The graph (e) shows the significant increase of choroidal thickness (mean ± SD) in patients with Graves' orbitopathy (*n* = 18) versus controls (*n* = 18) (^∗^*p* < 0.01).

The subfoveal choroid was significantly thicker in GO than in control eyes (399.2 ± 84.1 *μ*m versus 344.5 ± 88.1 *μ*m, respectively; *F* = 9.6, *p* = 0.003, adjusted for age, gender, axial length, and smoking) (Figure 3). The interexaminer correlation coefficients for the subfoveal choroidal thickness measurements were 0.98 (95% CI, 0.97–0.99).

Correlation analysis in the GO group showed a significant direct correlation between choroidal thickness and disease activity score (CAS) (*r* = 0.40, *p* = 0.02) as well as with the proptosis assessment with Hertel exophthalmometry (*r* = 0.36, *p* = 0.03). In patients with CAS ≥ 3, choroidal thickness was significantly increased than in the group with CAS < 3 (436.2 ± 97.5 *μ*m versus 369.6 ± 89.3 *μ*m, respectively, *p* = 0.02). Furthermore, choroidal thickness was negatively correlated with disease duration (*r* = −0.43, *p* = 0.008). No significant correlation was observed between choroidal thickness and habit of smoking, diplopia, or severity grading of GO.

## 4. Discussion

In this study, using newer generation, high-resolution, spectral domain OCT and EDI technique, we noninvasively assessed choroidal thickness change in a cohort of 18 GO patients. This study confirmed the previous studies reporting a significant increase in choroidal thickness in patients with GO when compared with healthy controls. In line with the other reports, we also found that choroidal thickness correlates with the clinical activity of GO (CAS score). In contrast with Ozkan et al., our data showed that the higher choroidal thickness in GO patients was significantly related with more severe proptosis. This finding may reflect the hemodynamics alterations of ophthalmic veins associated with orbital congestion. Indeed, increasing evidence suggests that ophthalmic vein congestion plays a significant role in the pathogenesis of the active stage of GO [9–11, 22]. A venous stasis has been described in several previous studies using color Doppler imaging, and a negative correlation has been found between orbital blood flow parameters and the clinical activity scores of the ocular diseases [23]. The choroidal thickening found in our series is probably due to a reduced choroidal drainage in the ophthalmic veins and, similar to venous stasis, it correlates with the disease activity, including the degree of proptosis.

In our results, we further found a negative correlation between choroidal thickness and disease duration suggesting an early involvement of the choroid in the natural history of GO.

Hence, it is reasonable to speculate that choroidal thickness measurement in patients with GO may be used as an indirect parameter to estimate the degree of orbital congestion, especially in those patients with subclinical and early GO manifestations. It is known that orbital color Doppler imaging (CDI) is characterized by several limitations that may affect orbital congestion (i.e., difficulties in detecting retrobulbar vessels, the pressure applied on the globe may decrease flow velocity, and minimal lid and eye movements may cause artificial color noise) [23, 24]. In contrast, OCT is a noninvasive, no contact technique that may potentially overcome these aforementioned CDI-related limits.

The main limitations of the present study are the small sample size and that choroidal analysis was based on subjective, nonautomated measurements. Further investigations are needed to establish the diagnostic and prognostic role of OCT analysis of choroidal thickness in appropriate long-term follow-up of a larger GO population.

## 5. Conclusion

In conclusion, our results suggest a potential role of OCT choroidal thickness measurement in estimating the degree of orbital congestion in GO. In fact, choroidal thickness was significantly higher in patients with active and early GO and higher proptosis values. The noninvasive, no contact imaging modality of OCT is easily accessible and may enable the clinician to detect the retrobulbar GO involvement, even in those patients with subclinical manifestations and/or at the beginning of the disease.

## Figures and Tables

**Table 1:** GO patients versus controls: demographics and baseline clinical characteristics.

Variable	GO patients	Controls	*p* value
Age (years)	44.1 ± 9.8	44.2 ± 10.7	1.0^∗^
Gender (male/female)	8/10	7/11	1.0^§^
Axial length (mm)	23.9 ± 0.8	24.0 ± 1.4	0.7^∗^
Spherical equivalent (diopters)	−0.6 ± 1.3	−0.4 ± 1.7	0.5°
Intraocular pressure (mmHg)	14.0 ± 1.9	13.4 ± 1.6	0.2°
BCVA (number of ETDRS letters)	61.9 ± 2.3	62.3 ± 2.1	0.4^∗^
Glycaemia (mg/dL)	80.8 ± 5.6	82.4 ± 6.3	0.4^∗^
LDL cholesterol (mg/dL)	82.2 ± 12.4	81.9 ± 11.9	0.9^∗^
Total cholesterol (mg/dL)	139.6 ± 13.3	132.2 ± 12.9	0.1^∗^
Systolic blood pressure (mmHg)	123.9 ± 7.1	122.8 ± 6.7	0.6°
Diastolic blood pressure (mmHg)	71.6 ± 4.2	70.8 ± 4.3	0.6°
Smoking/no smoking	12/6	9/9	0.5^§^

Values are mean ± SD unless otherwise indicated. ^∗^Unpaired *t*-test with Levene's test for equality of variances. °Mann–Whitney *U* test. ^§^Fisher's exact test.

## References

[1] Brent G. A. (2008). Graves’ disease. *The New England Journal of Medicine*.

[2] Bahn R. S. (2010). Graves’ ophthalmopathy. *The New England Journal of Medicine*.

[3] Orgiazzi J., Wiersinga W. M., Kahaly G. J. (2007). Pathogenesis. *Graves’Orbitopathy: A Multidisciplinary Approach*.

[4] Bahn R. S. (2002). Thyrotropin receptor expression in orbital adipose/connective tissues from patients with thyroid-associated ophthalmopathy. *Thyroid*.

[5] Bruscolini A., Abbouda A., Locuratolo N., Restivo L., Trimboli P., Romanelli F. (2015). Dry eye syndrome in non-exophthalmic Graves’ disease. *Seminas in Ophthalmology*.

[6] Smith T. J. (2010). Pathogenesis of Graves’ orbitopathy: a 2010 update. *Journal of Endocrinological Investigation*.

[7] Bartalena L., Tanda M. L. (2009). Clinical practice. Graves’ ophthalmopathy. *New England Journal of Medicine*.

[8] Saber E., McDonnell J., Zimmermann K. M., Yugar J. E., Feldon S. E. (1996). Extraocular muscle changes in experimental orbital venous stasis: some similarities to Graves’ orbitopathy. *Graefe's Archive for Clinical and Experimental Ophthalmology*.

[9] Alp M. N., Ozgen A., Can I., Cakar P., Gunalp I. (2000). Colour Doppler imaging of the orbital vasculature in Graves’ disease with computed tomographic correlation. *British Journal of Ophthalmology*.

[10] Somer D., Ozkan S. B., Ozdemir H., Atilla S., Söylev M. F., Duman S. (2002). Colour Doppler imaging of superior ophthalmic vein in thyroid-associated eye disease. *Japanese Journal of Ophthalmology*.

[11] Monteiro M. L. R., Angotti-Neto H. A., Benabou J. E., Betinjane A. J. (2008). Color Doppler imaging of the superior ophthalmic vein in different clinical forms of Graves’ orbitopathy. *Japanese Journal of Ophthalmology*.

[12] Çalışkan S., Acar M., Gürdal C. (2017). Choroidal thickness in patients with Graves’ ophthalmopathy. *Current Eye Research*.

[13] Özkan B., Koçer Ç. A., Altintaş Ö., Karabaş L., Acar A. Z., Yüksel N. (2016). Choroidal changes observed with enhanced depth imaging optical coherence tomography in patients with mild graves orbitopathy. *Eye*.

[14] Margolis R., Spaide R. F. (2009). A pilot study of enhanced depth imaging optical coherence tomography of the choroid in normal eyes. *American Journal of Ophthalmology*.

[15] Nickla D. L., Wallman J. (2010). The multifunctional choroid. *Progress in Retinal and Eye Research*.

[16] Tan K. A., Gupta P., Agarwal A. (2016). State of science: choroidal thickness and systemic health. *Survey of Ophthalmology*.

[17] Ulaş F., Doğan U., Duran B., Keleş A., Ağca S., Çelebi S. (2013). Choroidal thickness changes during the menstrual cycle. *Current Eye Research*.

[18] Mourits M. P., Prummel M. F., Wiersinga W. M., Koornneef L. (1997). Clinical activity score as a guide in the management of patients with Graves’ ophthalmopathy. *Clinical Endocrinology*.

[19] Bartalena L., Baldeschi L., Dickinson A. (2008). Consensus statement of the European Group on Graves’ orbitopathy (EUGOGO) on management of GO. *European Journal of Endocrinology*.

[20] Gharbiya M., Trebbastoni A., Parisi F. (2014). Choroidal thinning as a new finding in Alzheimer’s disease: evidence from enhanced depth imaging spectral domain optical coherence tomography. *Journal Alzheimers Disease*.

[21] Häner N. U., Dysli M., Abegg M., Zinkernagel M. S. (2015). Enhanced-depth optical coherence tomography for imaging horizontal rectus muscles in Graves’ orbitopathy. *Graefe's Archive for Clinical and Experimental Ophthalmology*.

[22] Walasik-Szemplińska D., Pauk-Domańska M., Sanocka U., Sudoł-Szopińska I. (2015). Doppler imaging of orbital vessels in the assessment of the activity and severity of thyroid-associated orbitopathy. *Journal of Ultrasonography*.

[23] Yanik B., Conkbayir I., Acaroglu G., Hekimoglu B. (2005). Graves’ ophthalmopathy: comparison of the Doppler sonography parameters with the clinical activity score. *Journal of Clinical Ultrasound*.

[24] Tranquart F., Bergès O., Koskas P. (2003). Color Doppler imaging of orbital vessels: personal experience and literature review. *Journal of Clinical Ultrasound*.

